# Short-Term and Long-Term COVID-19 Pandemic Forecasting Revisited with the Emergence of OMICRON Variant in Jordan

**DOI:** 10.3390/vaccines10040569

**Published:** 2022-04-07

**Authors:** Tareq Hussein, Mahmoud H. Hammad, Ola Surakhi, Mohammed AlKhanafseh, Pak Lun Fung, Martha A. Zaidan, Darren Wraith, Nidal Ershaidat

**Affiliations:** 1 Environmental and Atmospheric Research Laboratory (EARL), Department of Physics, School of Science, The University of Jordan, Amman 11942, Jordan; mhm0179430@ju.edu.jo; 2 Institute for Atmospheric and Earth System Research (INAR/Physics), University of Helsinki, FI-00014 Helsinki, Finland; pak.fung@helsinki.fi (P.L.F.); or martha.zaidan@nju.edu.cn (M.A.Z.); 3 Computer Science Department, Faculty of Information Technology, Middle East University, Amman 11831, Jordan; osurakhi@meu.edu.jo; 4 Department of Computer Science, Birzeit University, West Bank P.O. Box 14, Palestine; malkhanafseh@birzeit.edu; 5 Joint International Research Laboratory of Atmospheric and Earth System Sciences, School of Atmospheric Sciences, Nanjing University, Nanjing 210023, China; 6 School of Public Health and Social Work, Queensland University of Technology, Brisbane 4000, Australia; d.wraith@qut.edu.au; 7 Department of Physics, School of Science, The University of Jordan, Amman 11942, Jordan; n.ershaidat@ju.edu.jo

**Keywords:** linear forecast, short/long-term forecast, hybrid forecast (HF), herd immunity

## Abstract

Three simple approaches to forecast the COVID-19 epidemic in Jordan were previously proposed by Hussein, et al.: a short-term forecast (STF) based on a linear forecast model with a learning database on the reported cases in the previous 5–40 days, a long-term forecast (LTF) based on a mathematical formula that describes the COVID-19 pandemic situation, and a hybrid forecast (HF), which merges the STF and the LTF models. With the emergence of the OMICRON variant, the LTF failed to forecast the pandemic due to vital reasons related to the infection rate and the speed of the OMICRON variant, which is faster than the previous variants. However, the STF remained suitable for the sudden changes in epi curves because these simple models learn for the previous data of reported cases. In this study, we revisited these models by introducing a simple modification for the LTF and the HF model in order to better forecast the COVID-19 pandemic by considering the OMICRON variant. As another approach, we also tested a time-delay neural network (TDNN) to model the dataset. Interestingly, the new modification was to reuse the same function previously used in the LTF model after changing some parameters related to shift and time-lag. Surprisingly, the mathematical function type was still valid, suggesting this is the best one to be used for such pandemic situations of the same virus family. The TDNN was data-driven, and it was robust and successful in capturing the sudden change in +qPCR cases before and after of emergence of the OMICRON variant.

## 1. Introduction

After the outbreak of the severe Acute Respiratory Syndrome Coronavirus 2 (SARS-CoV-2), which was by the end of year 2019, many models have been developed to forecast/predict the Coronavirus Disease (COVID-19) epidemic temporal variation. Some of these models were based on Artificial Intelligence (AI) and Machine Learning (ML) or to some extent based on a simple mathematical model [1,2,3]. Modelling is an effective tool in studying the qualitative properties and dynamical behaviors of different diseases [4,5,6], and especially in improving the prognostic processes of COVID-19. Nevertheless, these models have several limitations, including reporting quality, understanding of factors related to social and clinical measures, slow development of spatial risk maps, and vaccination strategies [7,8,9,10,11,12,13]. Ultimately, some of these predictive models were utilized for limiting the spread of COVID-19 in an intervention of optimized strategies [14,15,16,17,18,19]. They can also be useful in developing public health measures to contain the spread of the virus.

There have been several attempts at modeling the COVID-19 pandemic outbreak in Jordan during the early stage of the COVID-19 pandemic outbreak [20,21,22,23]. However, they were limited to the lockdown period and did not span a long enough time to cover the alternating waves of outbreaks, making them outdated to handle the complete picture of the COVID-19 epidemic over a long-term period. Besides this, they did not include vaccination scenarios on limiting the COVID-19 infection rates. As one of the disadvantages, these models were complicated and difficult to be interpreted. Recently, Hussein, et al. [1] proposed three simple approaches to forecast the COVID-19 epidemic in Jordan; see also Section S1 in Supplementary Materials:

1. Short-term forecast (STF) model;
2. Long-term forecast (LTF) model;
3. Hybrid forecast (HF) model.

As will be illustrated in the next section, the STF and the HF suggest approaches fairly predicting the COVID-19 pandemic curve in Jordan. The LTF approach deviated from the real epi curve, and this might be explained when taking into account herd immunity and the vaccination trend in Jordan. However, with the emergence of the OMICRON variant, the LTF totally failed to forecast the COVID-19 pandemic due to a vital reason related to infection rate and the speed of the OMICRON variant, which is faster than the previous variants. However, the STF remained capable of predicting the sudden changes in the epi curve, because these simple models learn from the previous data of reported cases. This required a revisit for the suggested approaches to include the emergence of the OMICRON variation in Jordan.

In this study, we aim at introducing a simple modification for the LTF and the HF models, which were previously published by Hussein, et al. [1], in order to better forecast the COVID-19 pandemic by considering the OMICRON variant. A time-delay neural network (TDNN) to model the data of the +qPCR cases before and after of emergence of the OMICRON variant is also proposed.

## 2. Materials and Methods

### 2.1. Reported COVID-19 Pandemic Data in Jordan

**Figure 1:**
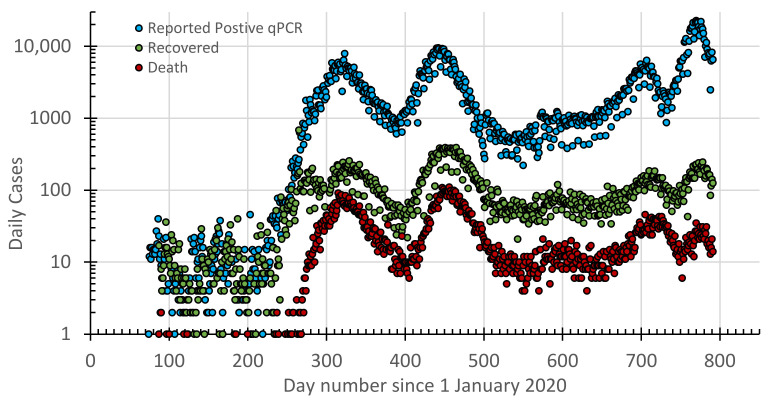
Timelines for the reported daily cases of +qPCR tests, those recovered, and deaths since 14 March 2020 in Jordan.

**Figure 2:**
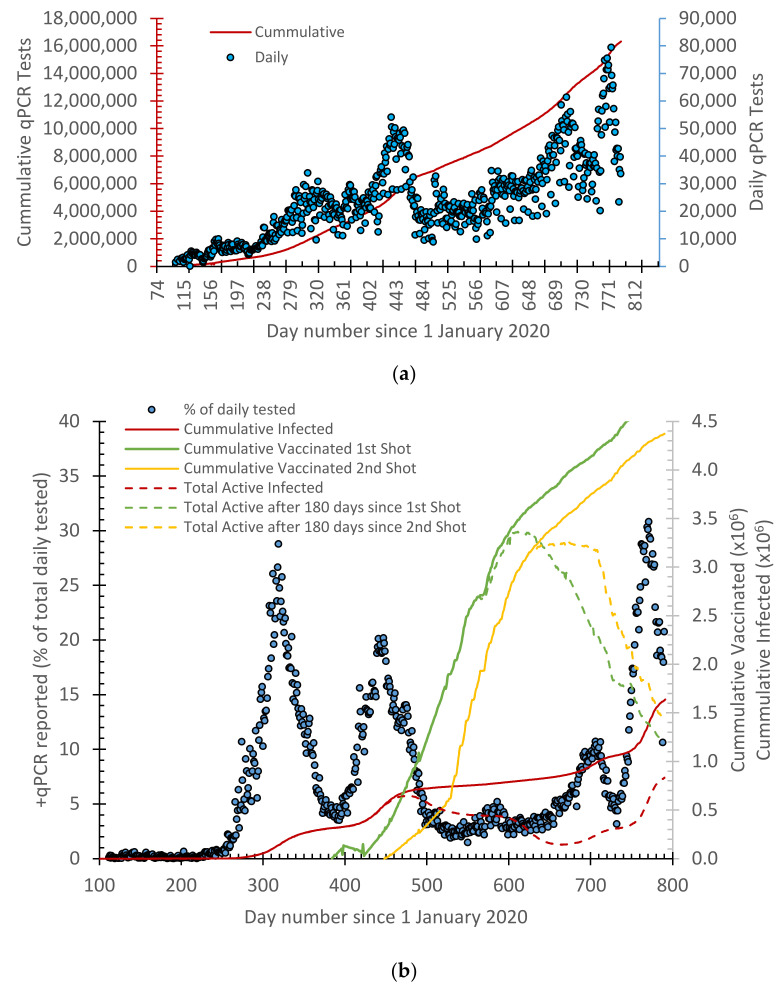
(**a**) qPCR tests performed in Jordan since 14 March 2020 and (**b**) percentage +qPCR cases overlayed with cumulative curves for the vaccination (first and second shots), as well as active cases representing herd immunity.

A COVID-19 database was cumulated from daily official reports by the Ministry of Health in Jordan. This included the daily reported positive cases of the qPCR tests, recovered cases, deaths, and vaccination shots (Figure 1 and Figure 2).

### 2.2. A Modified Model to Describe the OMICRON Variant

In order to include the OMICRON outbreak, we followed a similar approach in defining a LTF model as the one in Equation (S2) (Section S1 in Supplementary Materials) with the parameters *A*, *a*, *b*, *δ*, and *c* defined as 185, −0.003, 9.01, 1080, and 0.07, respectively. This is described by Hussein, et al. [1]. The STF model was unchanged, and the HF model was set up as described in Section S1 in Supplementary Materials [1].

### 2.3. Time Delay Neural Network (TDNN)

Time Delay Neural Network (TDNN) is a general class of dynamic networks that is well suited to deal with the time-series problems and temporal dependencies in large and small data. TDNN is similar to the feed-forward networks, but the input weight in the TDNN has a delay associated with it. In the regular feed-forward network, the input is multiplied by the weights, then passed to the nonlinear activation function such as the sigmoid function to produce the output layer. In the TDNN, a delay interval from D_1_ to D_n_ is added to the current input, then the input is multiplied by a specific weight to be compared with the past history of events, and this makes the input layer dynamic [24,25,26]. The output of the TDNN is estimated through the activation function like a regular feed-forward neural network to optimize the network weights.

In this case, TDNN is used to predict the +qPCR cases reported in Jordan, $\hat{y}$, through the function of the previous number of cases, *f(x,w)*, to optimize the weight, *w*. The architecture of the network is composed of one input layer, one hidden layer, and one output layer. In order to attain the time delay, the input layer uses a delay component embedded in the input units. The number of these delay components used in the network to give the best output estimation is still a challenging issue to be resolved. More details about the utilized TDNN model are found in Section S2 in the Supplementary Materials.

## 3. Results and Discussion

### 3.1. The History of the COVID-19 Pandemic in Jordan

In Jordan, the COVID-19 epidemic consisted of three waves before the OMICRON variant. These waves are summarized in Table 1. The first case was reported on 14 March 2020. After that, the Jordanian government took a series of immediate actions (“lockdown” and “curfew”) to limit the spread of SARS-COV-2. The first wave started in late September 2020, reaching its maximum in the middle of November 2020 with about 29% +qPCR cases. This first wave was over by mid-January 2021, when the +qPCR testes were as low as 4%. The second wave triggered in early February 2021, reaching its maximum around mid-March 2021, and was over in mid-May 2021, with about 20% +qPCR cases reported. A minor wave, which is referred to here as the undeveloped third wave, was reported in the first week of August 2021, with maximum +qPCR cases reported at about 5%. This undeveloped wave was over around mid-November 2021. The fourth wave was reported around mid-December 2021 with maximum +qPCR tests around 11%. This wave started to decline, reaching about 3% +qPCR tests by New Year’s Eve. These waves are summarized in Table 1.

The previously described waves were reported as outbreaks of the COVID-19 variations before the OMICRON variant. The first cases of the OMICRON variant were reported in Jordan during early January 2022, when the epi curve climbed aggressively, reporting the fifth wave with a maximum during the first week of February 2022, reaching about 31% +qPCR tests.

Interestingly, the first two waves as well as the undeveloped wave were separated by 6 weeks, and each wave spanned about three months (Figure 2). The fourth wave did not develop, reaching its end because the OMICRON variant wave kicked in and overlapped with it.

Reading the vaccination and infection curves (Figure 2b), clearly suggests that vaccination and herd immunity had an effect on lowering (i.e., weakening) the second wave and, to some extent, the non-development of the third wave. However, the vaccination trend slowed down, and people’s awareness decreased, which led to an aggressive emergence of the OMICRON wave in January 2022. While reporting this here, the OMICRON wave has not yet reached its end.

### 3.2. A Modified Forecast Model to Describe the OMICRON Variant Wave

**Figure 3:**
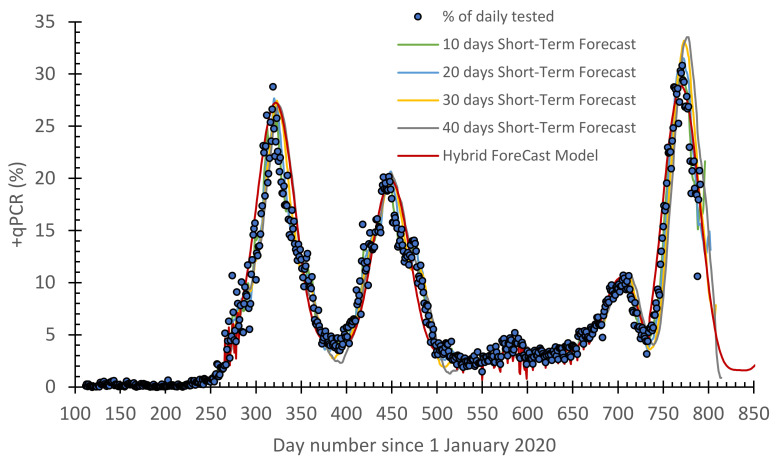
A timeline for the daily reported +qPCR tests overlayed by the short-term forecast (STF, with 10–40 days learning) model and compared with the hybrid forecast (HF) model for the COVID-19 pandemic in Jordan.

**Figure 4:**
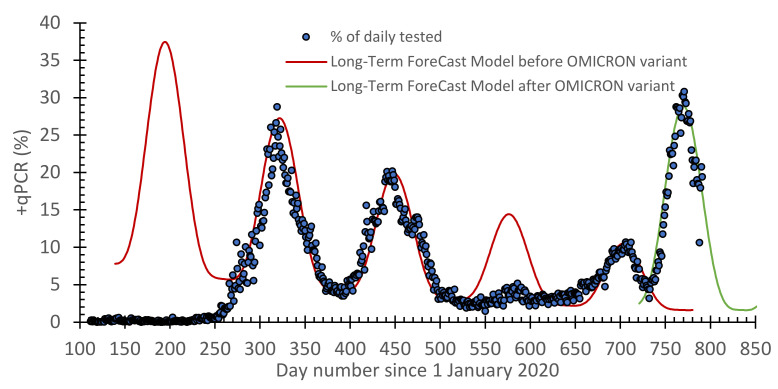
A timeline for the daily reported +qPCR tests overlayed by the long-term forecast (LTF) model predictions for the COVID-19 pandemic in Jordan before and after the emergence of the OMCIRON variant.

As previously noted, the best STF model was the one based on the learning period as 10–20 days (Figure 3). Since the STF model has a fast response, it is capable of predicting the OMCIRON wave. As for the LTF model (Figure 4), the selected function is still capable of forecasting the reported COVID-19 cases before and after the OMCIRON variant. It only needed the parameters *A*, *a*, *b*, *δ*, and *c* in Equation (S2) (Section S1 in Supplementary Materials) to be redefined. After a slight modification, the HF model is still capable of forecasting the epidemic. This will be shown later in this section.

### 3.3. Time Delay Neural Network (TDNN)

**Figure 5:**
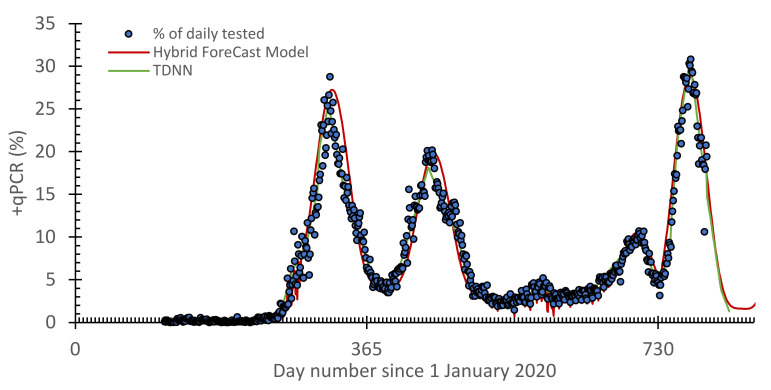
Comparison between the time delay neural network (TDNN) model and the hybrid forecast (HF) model for the +qPCR cases.

The results obtained from the training TDNN to model positive cases in Jordan are given in Figure 5 with a comparison with the HF model. The performance metrics of modeling using positive cases in Jordan were tested according to *R*^2^, *RMAE*, and *MSE*. The performance values are given in Table 2. Quantitatively, these were 0.97, 1.2, and 0.7 for *R*^2^, *RMAE*, and *MSE*, respectively.

The analysis of the results shows that the forecasting model, TDNN, made an accurate prediction despite changing the statistical properties of the target data (with the appearance of the Omicron version) over time. The data-driven models can be a valuable tool to understand and analyze phenomena (such as COVID-19) and make predictions.

**Figure 6:**
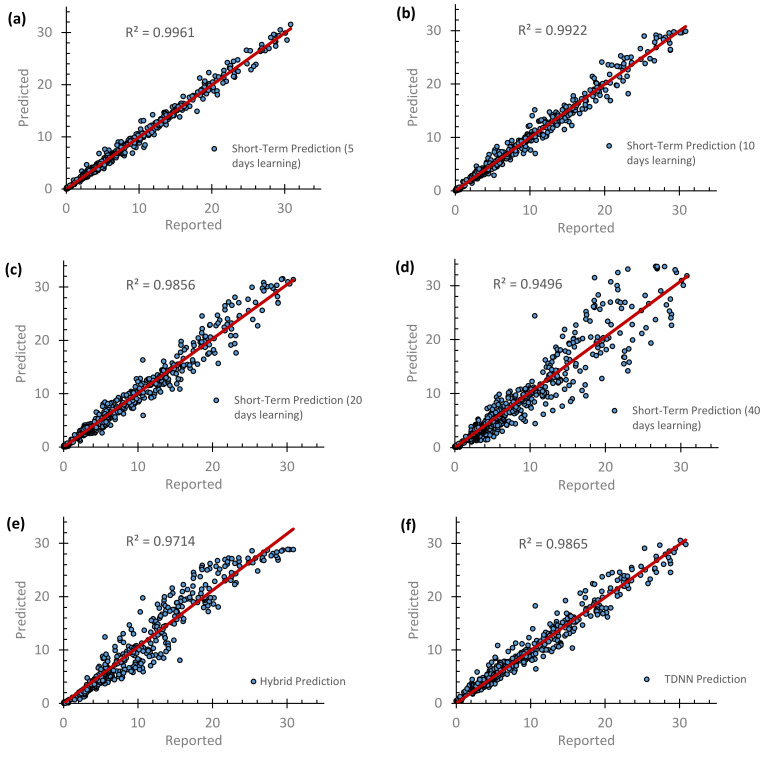
Comparisons of the forecasted versus the reported +qPCR daily cases, including the periods before and after the emergence of the OMICRON variant, using (**a**–**d**) STF model prediction that takes 5, 10, 20 and 40 days of learning, (**e**) HF model prediction and (**f**) TDNN prediction.

After all, the scatter plots for the predicted −qPCR daily cases against the reported ones are presented in Figure 6. Together with the performance of the models (i.e., Table 2), this shows that increasing the number of learning days reduced the *R*^2^ value and also the performance of the STF model. However, a five-days learning approach should be avoided because it is not realistic, as it replicates the reported data. Taking 40 days gives a time-lag in the forecasted data. As such, a reasonable learning period can be 10–20 days. Interestingly, the selected mathematical function of the LTF model, which is also impeded in the HF model, is capable of forecasting the reported COVID-19 cases before the emergence of the OMICRON variation. However, the same function was still valid with a slight change to its parameters. The overall performance of the HF model was 0.95, 1.9, and 1.1 for *R*^2^, *RMAE*, and *MSE*, respectively.

## 4. Conclusions

Herein, a simple prediction approach was proposed to forecast COVID-19 reported cases in Jordan for a trail to include the OMICRON variant. The basic principle was built on our previous approaches [1]: short-term forecast (STF) model, long-term forecasting (LTF) model, and hybrid approach. In the end, the best model was the TDNN, followed by the HF model that included the LTF models before and after the OMICRON variant outbreak. When the LTF model failed to cope with the sudden temporal variation of the pandemic, the parameters of the STF model were reconsidered. The outcomes of this investigation can be applied to COVID-19 pandemic cases that are similar to the Jordanian case.

## Figures and Tables

**Table 1:** A summary about the pandemic waves in Jordan.

Wave	Start	End	Peak
First ^1^	September 2020	Mid-January 2021	Mid-November 2020
Second ^2^	February 2021	Mid-May 2021	Mid-March 2021
Third ^3^	August 2021	Mid-November 2021	Mid-August 2021
Fourth ^4^	December 2021	New Year Eve	Mid-December 2021

^1^ Peak value was about 29% +qPCR cases, and lowest value was as low as 4%. ^2^ Peak value was about 20% +qPCR cases, and lowest value was as low as 2%. ^3^ Undeveloped wave with peak value at about 5% +qPCR cases, and lowest value was as low as 2%. ^4^ Uncompleted wave with peak value at about 11% +qPCR cases, and it never reached its minimum, as the OMICRON variant wave started and overlapped with the end of this fourth wave.

**Table 2:** Evaluation metrics of the three forecasting models: short-term forecast (STF), hybrid forecast (HF), and time delay neural network (TDNN) model for the whole COVID-19 pandemic thus far in Jordan, including before and after the emergence of OMICRON variant.

Model	Number of Learning Days	*R* ^2^	*RMSE*	*MAE*
STF	5	0.99	0.62	0.37
	10	0.98	0.87	0.52
	15	0.98	1.04	0.65
	20	0.97	1.22	0.75
	25	0.96	1.47	0.88
	30	0.94	1.77	1.04
	35	0.93	2.07	1.19
	40	0.90	2.35	1.33
HF		0.95	1.89	1.09
TDNN		0.97	1.15	0.74

## Data Availability

Data is available upon request.

## Supplementary Materials

The following supporting information can be downloaded at: https://www.mdpi.com/article/10.3390/vaccines10040569/s1, Section S1. Forecast Models for the Postive qPCR Tests. Figure S1. Scheme showing the short-term forecast (STF) model. This Figure was adopted from Hussein, et al. [1]. Figure S2. A scheme showing the long-term forecast (LTF) model. This Figure was adopted from Hussein, et al. [1]. Figure S3. A scheme showing the hybrid forecast (HF) model. This Figure was adopted from Hussein, et al. [1]. Section S2. Time Delay Neural Network (TDNN). FigureS4: The time-delay neural network (TDNN) architecture.
