# Supplementary material for: Short-Term and Long-Term COVID-19 Pandemic Forecasting Revisited with the Emergence of OMICRON Variant in Jordan
Source: Vaccines (Basel). 2022 Apr 7;10(4):569. doi: 10.3390/vaccines10040569 (PMC9025683; doi:10.3390/vaccines10040569)
Supplement: Supplementary file 1 [file vaccines-10-00569-s001.zip › vaccines-1655471-supplementary.pdf]

vaccines

Supplementary Material

Short-Term and Long-Term COVID-19 Pandemic Forecasting Revisited with the Emergence of OMICRON Variant in Jordan

Hussein et al.

S1. Forecast Models for the Postive qPCR Tests

The short-term forecast (STF) model was based on a simple linear forecast model (built-in function "*forecast.linear*" in Microsoft® Excel, Microsoft® office 365, Figure S1)

$$Y = forecast.linear(X,x,y), \tag{S1}$$

where Y is the predicted percentage of positive qPCR test(s) on day(s) X. The learning data are the time range (x) and corresponding observed (i.e., reported) positive qPCR tests (y). The range of x and y spans over the previous days (between 5 and 40 days); i.e., the dataset (x,y) is the learning data for the linear prediction of Y on day X. The Microsoft Excel *forecast.linear*(X,x,y) function predicts a value based on existing values along a linear trend.

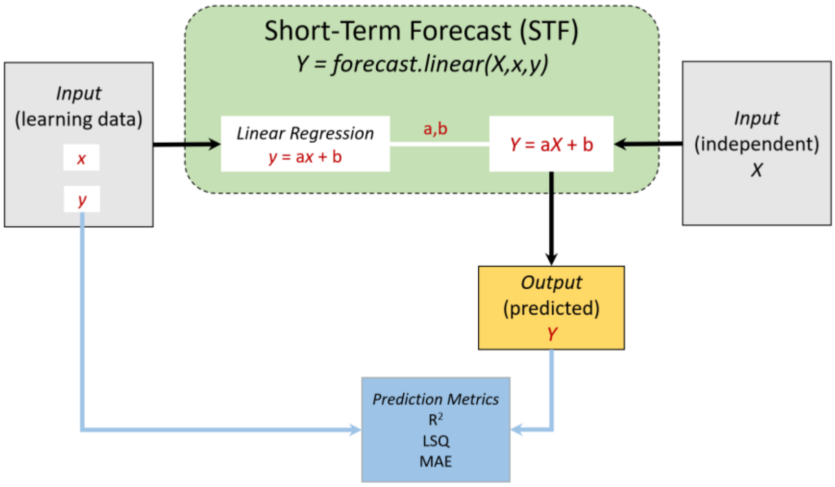

Figure S1. Scheme showing the short-term forecast (STF) model. This Figure was adopted from Hussein et al. [1].

The long-term forecast (LTF) model was based on a white-box approach by choosing the best mathematical function that describes the previously reported percentage of positive daily qPCR tests (as percentage out of the total daily qPCR tests); Figure S2. A preliminary and exploratory analysis for this database indicated that the best fit has the following mathematical form

$$Y = Ae^{-aX}\left(cos^4\left(\frac{bX+\delta}{365}\right)+c\right) \tag{S2}$$

where Y is the predicted percentage of positive qPCR tests on day X (as day number after 01.01.2020). The parameters A, a, b, δ, and c are the average model parameters that best fit this function with the database and are presented in Figure 9. These were 50, -0.0025, 9.01, 530, and 0.22, respectively. This function was chosen in analogy with the concepts of some physical phenomena (e.g., damping oscillator) after a slight modification to the power of the trigonometric function.

The hybrid forecast (HF) model was suggested to be a combination between the STF and the LTF models (Figure S3) based on the following criteria:

- During curfew and lockdown periods, the HF was applied
- During normal life condition periods, the LTF model was applied

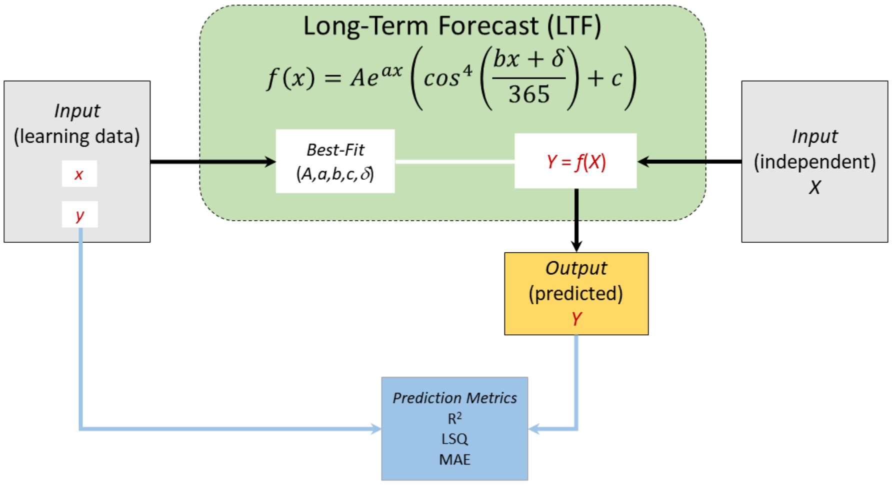

Figure S2. A scheme showing the long-term forecast (LTF) model. This Figure was adopted from Hussein et al. [1].

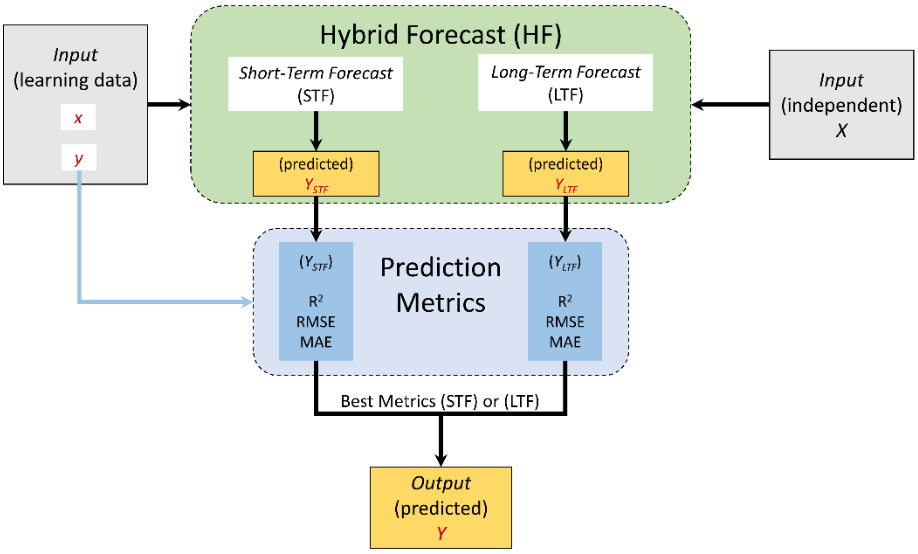

Figure S3. A scheme showing the hybrid forecast (HF) model. This Figure was adopted from Hussein et al. [1].

The prediction accuracy was tested by the coefficient of determination (R^2), root mean square error (RMSE) and mean absolute error (MAE), as follows:

$$R^2 = 1 - \frac{\sum_{i=1}^{n}(\hat{y}_i - y_i)^2}{\sum_{i=1}^{n}(y_i - \bar{y})^2}, \tag{S3}$$

$$RMSE = \sqrt{\frac{\sum_{i=1}^{n}(\hat{y}_i - y_i)^2}{n}}, \tag{S4}$$

$$MAE = \frac{\sum_{i=1}^{n}|\hat{y}_i - y_i|}{n}, \tag{S5}$$

where y_i and $\hat{y}_i$ represent, respectively, the ith observed value and the ith predicted value. $\bar{y}$ denotes the mean of the observed dataset of n data points. R^2 is a measure the linear association between the observed variable and the predicted output variable by the selected model. The higher the value of R^2, the higher the variability of the predicted variable the model can explain. While both *RMSE* and *MAE* measure the average difference between the observed and the predicted variable, the difference between these two terms lies in that *RMSE* represents the quadratic mean of these differences, yet *MAE* calculates the absolute difference.

S2. Time Delay Neural Network (TDNN)

The TDNN is well suited to deal with time-series problems and temporal dependencies in large and small data. Here, a pair of the current and previous value (x_t ,x_{t-1}) was used to predict the future value x_{t+1} through the running of a non-linear sigmoid activation function to find the relationship between inputs and output. The weights of the network were adjusted through the backpropagation algorithm and the number of epochs for training the algorithm was set to 3000 epochs. Figure S4 shows the architecture of the network.

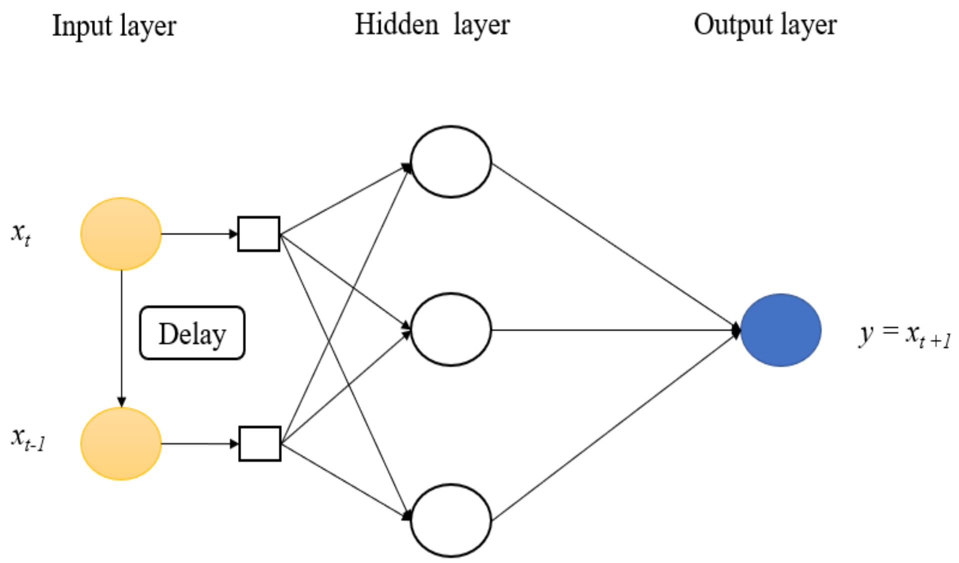

Figure S4. The time-delay neural network (TDNN) architecture.
